# (*E*)-4-Amino-*N*-(1,2-dihydro­pyridin-2-yl­idene)benzene­sulfonamide nitro­methane monosolvate

**DOI:** 10.1107/S1600536812009865

**Published:** 2012-03-10

**Authors:** Mostafa M. Ghorab, Mansour S. Al-Said, Hazem A. Ghabbour, Suchada Chantrapromma, Hoong-Kun Fun

**Affiliations:** a Medicinal, Aromatic and Poisonous Plants Research Center (MAPPRC), College of Pharmacy, King Saud University, PO Box 2457, Riyadh 11451, Saudi Arabia; b Department of Pharmaceutical Chemistry, College of Pharmacy, King Saud University, PO Box 2457, Riyadh 11451, Saudi Arabia; c Crystal Materials Research Unit, Department of Chemistry, Faculty of Science, Prince of Songkla University, Hat-Yai, Songkhla 90112, Thailand; d X-ray Crystallography Unit, School of Physics, Universiti Sains Malaysia, 11800 USM, Penang, Malaysia

## Abstract

In the title solvate, C_11_H_11_N_3_O_2_S·CH_3_NO_2_, the dihedral angle between the benzene ring and the N-containing ring is 85.94 (11)°, and an approximate V shape arises for the sulfonamide mol­ecule. In the crystal, N—H⋯O and N—H⋯N hydrogen bonds and weak C—H⋯O inter­actions link the sulfonamide mol­ecules into a three-dimensional network. The nitro­methane solvent mol­ecules are located in the inter­stitial sites in the sulfonamide network.

## Related literature

For background to the applications of sulfonamide compounds, see: Ghorab *et al.* (2009 ▶);
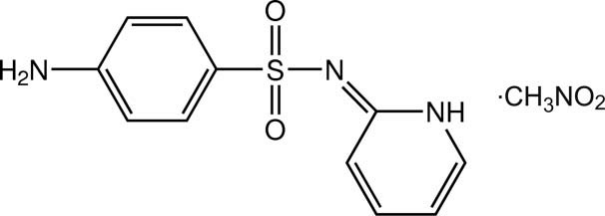


## Experimental

### 

#### Crystal data

C_11_H_11_N_3_O_2_S·CH_3_NO_2_
*M* *_r_* = 310.34
Orthorhombic, 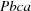

*a* = 10.5179 (3) Å
*b* = 12.4857 (3) Å
*c* = 22.7237 (6) Å
*V* = 2984.15 (14) Å^3^
*Z* = 8
Cu *K*α radiation
μ = 2.14 mm^−1^
*T* = 296 K
0.54 × 0.43 × 0.31 mm

#### Data collection

Bruker SMART APEXII CCD diffractometer
Absorption correction: multi-scan (*SADABS*; Bruker, 2009 ▶) *T*_min_ = 0.392, *T*_max_ = 0.554
14569 measured reflections
2799 independent reflections
2386 reflections with *I* > 2σ(*I*)
*R*_int_ = 0.062

#### Refinement

*R*[*F* ^2^ > 2σ(*F* ^2^)] = 0.045
*wR*(*F* ^2^) = 0.144
*S* = 1.07
2799 reflections
196 parameters
H atoms treated by a mixture of independent and constrained refinement
Δρ_max_ = 0.25 e Å^−3^
Δρ_min_ = −0.42 e Å^−3^

### 

Data collection: *APEX2* (Bruker, 2009 ▶); cell refinement: *SAINT* (Bruker, 2009 ▶); data reduction: *SAINT*; program(s) used to solve structure: *SHELXTL* (Sheldrick, 2008 ▶); program(s) used to refine structure: *SHELXTL*; molecular graphics: *SHELXTL*; software used to prepare material for publication: *SHELXTL* and *PLATON* (Spek, 2009 ▶).

## Supplementary Material

Crystal structure: contains datablock(s) global, I. DOI: 10.1107/S1600536812009865/hb6660sup1.cif

Structure factors: contains datablock(s) I. DOI: 10.1107/S1600536812009865/hb6660Isup2.hkl

Supplementary material file. DOI: 10.1107/S1600536812009865/hb6660Isup3.cml

Additional supplementary materials:  crystallographic information; 3D view; checkCIF report

## Figures and Tables

**Table 1:** Hydrogen-bond geometry (Å, °)

*D*—H⋯*A*	*D*—H	H⋯*A*	*D*⋯*A*	*D*—H⋯*A*
N2—H1*N*2⋯O2^i^	0.87	2.10	2.971 (3)	174
N2—H2*N*2⋯O1^ii^	0.87	2.34	3.162 (3)	157
N3—H1*N*3⋯N1^iii^	0.86 (2)	2.09 (2)	2.948 (2)	178 (2)
C8—H8*A*⋯O1^iii^	0.93	2.52	3.160 (3)	126

Symmetry codes: (i) 
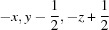
; (ii) 
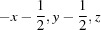
; (iii) 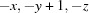.

**Fig. 1.**
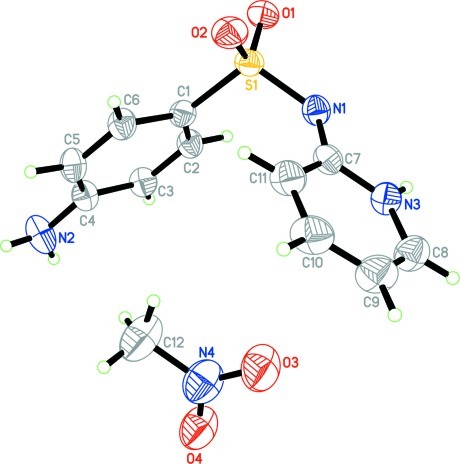
The structure of (I), showing 30% probability displacement ellipsoids.

**Fig. 2.**
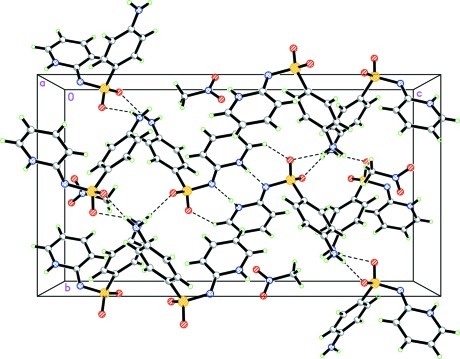
The crystal packing of (I) viewed along the a axis, showing three dimensional network.

## supplementary crystallographic information

### Comment

As part of our ongoing studies of sulfonamides with potential biological activities (Ghorab *et al.* (2009), the present investigation deals with the synthesis of the title compound, (I), (Fig. 1) a sulfonamide bearing a pyridine moiety to be evaluated as anticancer agent.

The environment of S atom is distorted tetrahedral geometry [angles around S atom are 104.49 (9) - 115.82 (10)°] with two O atoms, one N atom of the amide group and one C atom of the benzene ring. The dihedral angle between the benzene ring and the N atom-containing ring is 85.94 (11)°. The amino group is co-planar with its bound benzene ring with r.m.s. 0.0126 (2) Å for the seven non H atoms (C1–C6/N2).

In the crystal (Fig. 2), the molecules of sulfonamide derivative are linked by N—H···O and N—H···N hydrogen bonds and weak C···H···O interactions (Table 1) into a three dimensional network. The nitromethane solvent molecules are located in the interstitial sites of the sulfonamide network.

### Experimental

A mixture of (*E*)-3-(dimethylamino)-1-(thiophen-2-yl)prop-2-en-1-one (1.81 g, 0.01 mole) and sulfapyridine (2.49 g, 0.01 mole) in absolute ethanol (30 ml) was heated under reflux for 12 hr. The reaction mixture was filtered and pink blocks of (I) were obtained by the slow evaporation of an ethanol/nitromethane (3:1) solution at room temperature.

### Refinement

Amide H atom was located in a difference map and refined isotropically. The remaining H atoms were placed in calculated positions with d(N-H) = 0.87 Å, d(C-H) = 0.93 for aromatic, and 0.96 Å for CH_3_ atoms. The *U*_iso_ values were constrained to be 1.5*U*_eq_ of the carrier atom for methyl H atoms and 1.2*U*_eq_ for the remaining H atoms. A rotating group model was used for the methyl groups.

### Crystal data

**Table:**

C_11_H_11_N_3_O_2_S·CH_3_NO_2_	*F*(000) = 1296
*M**_r_* = 310.34	*D*_x_ = 1.381 Mg m^−^^3^
Orthorhombic, *P**b**c**a*	Cu *K*α radiation, λ = 1.54178 Å
Hall symbol: -P 2ac 2ab	Cell parameters from 2799 reflections
*a* = 10.5179 (3) Å	θ = 5.7–71.6°
*b* = 12.4857 (3) Å	µ = 2.14 mm^−^^1^
*c* = 22.7237 (6) Å	*T* = 296 K
*V* = 2984.15 (14) Å^3^	Block, pink
*Z* = 8	0.54 × 0.43 × 0.31 mm

### Data collection

**Table:**

Bruker SMART APEXII CCD diffractometer	2799 independent reflections
Radiation source: fine-focus sealed tube	2386 reflections with *I* > 2σ(*I*)
Graphite monochromator	*R*_int_ = 0.062
φ and ω scans	θ_max_ = 71.6°, θ_min_ = 5.7°
Absorption correction: multi-scan (*SADABS*; Bruker, 2009)	*h* = −11→10
*T*_min_ = 0.392, *T*_max_ = 0.554	*k* = −15→15
14569 measured reflections	*l* = −27→27

### Refinement

**Table:**

Refinement on *F*^2^	Secondary atom site location: difference Fourier map
Least-squares matrix: full	Hydrogen site location: inferred from neighbouring sites
*R*[*F*^2^ > 2σ(*F*^2^)] = 0.045	H atoms treated by a mixture of independent and constrained refinement
*wR*(*F*^2^) = 0.144	*w* = 1/[σ^2^(*F*_o_^2^) + (0.0843*P*)^2^ + 0.5181*P*] where *P* = (*F*_o_^2^ + 2*F*_c_^2^)/3
*S* = 1.07	(Δ/σ)_max_ = 0.001
2799 reflections	Δρ_max_ = 0.25 e Å^−^^3^
196 parameters	Δρ_min_ = −0.42 e Å^−^^3^
0 restraints	Extinction correction: *SHELXTL* (Sheldrick, 2008), Fc^*^=kFc[1+0.001xFc^2^λ^3^/sin(2θ)]^-1/4^
Primary atom site location: structure-invariant direct methods	Extinction coefficient: 0.0022 (3)

### Special details

**Table:**

Geometry. All esds (except the esd in the dihedral angle between two l.s. planes) are estimated using the full covariance matrix. The cell esds are taken into account individually in the estimation of esds in distances, angles and torsion angles; correlations between esds in cell parameters are only used when they are defined by crystal symmetry. An approximate (isotropic) treatment of cell esds is used for estimating esds involving l.s. planes.
Refinement. Refinement of F^2^ against ALL reflections. The weighted R-factor wR and goodness of fit S are based on F^2^, conventional R-factors R are based on F, with F set to zero for negative F^2^. The threshold expression of F^2^ > 2sigma(F^2^) is used only for calculating R-factors(gt) etc. and is not relevant to the choice of reflections for refinement. R-factors based on F^2^ are statistically about twice as large as those based on F, and R- factors based on ALL data will be even larger.

### Fractional atomic coordinates and isotropic or equivalent isotropic displacement parameters (Å^2^)

**Table:**

	*x*	*y*	*z*	*U*_iso_*/*U*_eq_	
S1	0.03202 (5)	0.52678 (4)	0.13912 (2)	0.0537 (2)	
O1	−0.04470 (16)	0.62229 (11)	0.13586 (7)	0.0664 (4)	
O2	0.14832 (16)	0.53613 (12)	0.17203 (7)	0.0696 (4)	
N1	0.05275 (16)	0.49231 (13)	0.07212 (8)	0.0549 (4)	
N2	−0.27661 (19)	0.18108 (17)	0.24298 (10)	0.0806 (6)	
H1N2	−0.2408	0.1417	0.2699	0.097*	
H2N2	−0.3364	0.1534	0.2213	0.097*	
N3	0.13565 (17)	0.39072 (14)	−0.00078 (8)	0.0587 (4)	
H1N3	0.082 (2)	0.4266 (17)	−0.0216 (10)	0.054 (6)*	
C1	−0.05963 (18)	0.42658 (14)	0.17206 (8)	0.0499 (4)	
C2	−0.1786 (2)	0.40208 (16)	0.14851 (9)	0.0547 (5)	
H2A	−0.2093	0.4405	0.1165	0.066*	
C3	−0.2504 (2)	0.32171 (17)	0.17237 (9)	0.0576 (5)	
H3A	−0.3299	0.3063	0.1565	0.069*	
C4	−0.20549 (19)	0.26212 (16)	0.22053 (9)	0.0567 (5)	
C5	−0.0891 (2)	0.29027 (18)	0.24482 (9)	0.0628 (5)	
H5A	−0.0599	0.2542	0.2780	0.075*	
C6	−0.0163 (2)	0.37042 (17)	0.22084 (9)	0.0576 (5)	
H6A	0.0623	0.3871	0.2373	0.069*	
C7	0.13465 (18)	0.41433 (15)	0.05728 (9)	0.0530 (4)	
C8	0.2136 (2)	0.3168 (2)	−0.02582 (12)	0.0741 (6)	
H8A	0.2104	0.3049	−0.0662	0.089*	
C9	0.2955 (3)	0.2609 (2)	0.00786 (14)	0.0855 (8)	
H9A	0.3498	0.2106	−0.0088	0.103*	
C10	0.2968 (3)	0.2799 (2)	0.06752 (14)	0.0857 (8)	
H10A	0.3523	0.2413	0.0913	0.103*	
C11	0.2185 (2)	0.35419 (18)	0.09265 (11)	0.0708 (6)	
H11A	0.2205	0.3651	0.1331	0.085*	
O3	0.0798 (3)	0.0856 (2)	0.04675 (11)	0.1239 (9)	
O4	−0.0699 (2)	−0.0317 (2)	0.06434 (11)	0.1120 (8)	
N4	0.0045 (3)	0.0378 (3)	0.08153 (15)	0.1093 (9)	
C12	0.0102 (4)	0.0684 (4)	0.14512 (17)	0.1265 (14)	
H12A	−0.0722	0.0921	0.1578	0.190*	
H12B	0.0706	0.1253	0.1503	0.190*	
H12C	0.0358	0.0076	0.1681	0.190*	

### Atomic displacement parameters (Å^2^)

**Table:**

	*U*^11^	*U*^22^	*U*^33^	*U*^12^	*U*^13^	*U*^23^
S1	0.0566 (4)	0.0492 (3)	0.0551 (3)	−0.00291 (18)	−0.00473 (19)	−0.00030 (17)
O1	0.0793 (11)	0.0487 (8)	0.0712 (10)	0.0061 (7)	0.0029 (7)	0.0001 (6)
O2	0.0653 (10)	0.0710 (9)	0.0724 (10)	−0.0133 (7)	−0.0152 (8)	−0.0063 (7)
N1	0.0541 (9)	0.0559 (8)	0.0546 (9)	0.0045 (7)	−0.0008 (7)	0.0066 (7)
N2	0.0624 (12)	0.0869 (13)	0.0923 (15)	−0.0077 (10)	−0.0068 (10)	0.0371 (11)
N3	0.0544 (10)	0.0631 (9)	0.0587 (10)	0.0083 (8)	−0.0006 (8)	0.0047 (8)
C1	0.0513 (10)	0.0518 (9)	0.0464 (9)	0.0002 (8)	−0.0059 (7)	−0.0004 (7)
C2	0.0549 (11)	0.0588 (10)	0.0505 (10)	0.0017 (8)	−0.0082 (8)	0.0071 (8)
C3	0.0479 (10)	0.0659 (11)	0.0589 (11)	−0.0003 (8)	−0.0077 (8)	0.0038 (9)
C4	0.0494 (10)	0.0626 (10)	0.0580 (11)	0.0039 (9)	0.0040 (8)	0.0082 (8)
C5	0.0618 (13)	0.0740 (12)	0.0525 (10)	0.0043 (10)	−0.0075 (9)	0.0153 (9)
C6	0.0546 (11)	0.0663 (11)	0.0518 (11)	−0.0017 (9)	−0.0110 (8)	0.0050 (8)
C7	0.0476 (10)	0.0520 (9)	0.0595 (11)	−0.0021 (8)	−0.0016 (8)	0.0072 (8)
C8	0.0688 (14)	0.0791 (14)	0.0744 (14)	0.0156 (12)	0.0052 (11)	−0.0043 (11)
C9	0.0784 (17)	0.0795 (15)	0.0985 (19)	0.0293 (14)	0.0006 (14)	−0.0035 (14)
C10	0.0832 (17)	0.0737 (14)	0.100 (2)	0.0258 (13)	−0.0213 (15)	0.0060 (13)
C11	0.0739 (15)	0.0672 (12)	0.0712 (14)	0.0110 (11)	−0.0150 (11)	0.0046 (10)
O3	0.1205 (18)	0.148 (2)	0.1029 (17)	−0.0638 (17)	0.0212 (14)	−0.0115 (15)
O4	0.0996 (15)	0.141 (2)	0.0955 (15)	−0.0502 (15)	0.0236 (13)	−0.0211 (13)
N4	0.0867 (17)	0.139 (2)	0.103 (2)	−0.0042 (17)	0.0109 (16)	−0.0112 (17)
C12	0.122 (3)	0.169 (4)	0.089 (2)	−0.013 (3)	0.004 (2)	−0.033 (2)

### Geometric parameters (Å, º)

**Table:**

S1—O2	1.4384 (16)	C5—C6	1.373 (3)
S1—O1	1.4418 (15)	C5—H5A	0.9300
S1—N1	1.5971 (18)	C6—H6A	0.9300
S1—C1	1.7477 (19)	C7—C11	1.410 (3)
N1—C7	1.343 (3)	C8—C9	1.347 (4)
N2—C4	1.358 (3)	C8—H8A	0.9300
N2—H1N2	0.8700	C9—C10	1.377 (4)
N2—H2N2	0.8699	C9—H9A	0.9300
N3—C7	1.352 (3)	C10—C11	1.365 (4)
N3—C8	1.359 (3)	C10—H10A	0.9300
N3—H1N3	0.86 (2)	C11—H11A	0.9300
C1—C6	1.389 (3)	O3—N4	1.268 (4)
C1—C2	1.395 (3)	O4—N4	1.232 (4)
C2—C3	1.368 (3)	N4—C12	1.496 (5)
C2—H2A	0.9300	C12—H12A	0.9600
C3—C4	1.405 (3)	C12—H12B	0.9600
C3—H3A	0.9300	C12—H12C	0.9600
C4—C5	1.388 (3)		
			
O2—S1—O1	115.82 (10)	C5—C6—C1	120.10 (19)
O2—S1—N1	113.66 (10)	C5—C6—H6A	119.9
O1—S1—N1	104.49 (9)	C1—C6—H6A	119.9
O2—S1—C1	107.73 (10)	N1—C7—N3	114.09 (17)
O1—S1—C1	107.78 (9)	N1—C7—C11	130.1 (2)
N1—S1—C1	106.90 (9)	N3—C7—C11	115.81 (19)
C7—N1—S1	121.49 (14)	C9—C8—N3	120.0 (2)
C4—N2—H1N2	116.5	C9—C8—H8A	120.0
C4—N2—H2N2	118.8	N3—C8—H8A	120.0
H1N2—N2—H2N2	119.1	C8—C9—C10	118.4 (2)
C7—N3—C8	124.15 (19)	C8—C9—H9A	120.8
C7—N3—H1N3	114.7 (15)	C10—C9—H9A	120.8
C8—N3—H1N3	121.1 (16)	C11—C10—C9	121.5 (2)
C6—C1—C2	119.35 (18)	C11—C10—H10A	119.2
C6—C1—S1	121.47 (15)	C9—C10—H10A	119.2
C2—C1—S1	119.17 (15)	C10—C11—C7	120.0 (2)
C3—C2—C1	120.26 (18)	C10—C11—H11A	120.0
C3—C2—H2A	119.9	C7—C11—H11A	120.0
C1—C2—H2A	119.9	O4—N4—O3	122.0 (3)
C2—C3—C4	120.77 (19)	O4—N4—C12	120.8 (3)
C2—C3—H3A	119.6	O3—N4—C12	117.2 (3)
C4—C3—H3A	119.6	N4—C12—H12A	109.5
N2—C4—C5	121.67 (19)	N4—C12—H12B	109.5
N2—C4—C3	120.14 (19)	H12A—C12—H12B	109.5
C5—C4—C3	118.18 (19)	N4—C12—H12C	109.5
C6—C5—C4	121.24 (18)	H12A—C12—H12C	109.5
C6—C5—H5A	119.4	H12B—C12—H12C	109.5
C4—C5—H5A	119.4		
			
O2—S1—N1—C7	43.60 (19)	C3—C4—C5—C6	−3.3 (3)
O1—S1—N1—C7	170.79 (16)	C4—C5—C6—C1	1.3 (3)
C1—S1—N1—C7	−75.12 (17)	C2—C1—C6—C5	1.2 (3)
O2—S1—C1—C6	−0.9 (2)	S1—C1—C6—C5	−178.33 (17)
O1—S1—C1—C6	−126.50 (18)	S1—N1—C7—N3	176.39 (14)
N1—S1—C1—C6	121.65 (17)	S1—N1—C7—C11	−3.6 (3)
O2—S1—C1—C2	179.59 (16)	C8—N3—C7—N1	178.0 (2)
O1—S1—C1—C2	53.96 (18)	C8—N3—C7—C11	−2.0 (3)
N1—S1—C1—C2	−57.89 (18)	C7—N3—C8—C9	0.8 (4)
C6—C1—C2—C3	−1.6 (3)	N3—C8—C9—C10	0.6 (4)
S1—C1—C2—C3	177.94 (16)	C8—C9—C10—C11	−0.6 (5)
C1—C2—C3—C4	−0.5 (3)	C9—C10—C11—C7	−0.7 (4)
C2—C3—C4—N2	−178.6 (2)	N1—C7—C11—C10	−178.1 (2)
C2—C3—C4—C5	2.9 (3)	N3—C7—C11—C10	1.9 (3)
N2—C4—C5—C6	178.2 (2)		

### Hydrogen-bond geometry (Å, º)

**Table:**

*D*—H···*A*	*D*—H	H···*A*	*D*···*A*	*D*—H···*A*
N2—H1*N*2···O2^i^	0.87	2.10	2.971 (3)	174
N2—H2*N*2···O1^ii^	0.87	2.34	3.162 (3)	157
N3—H1*N*3···N1^iii^	0.86 (2)	2.09 (2)	2.948 (2)	178 (2)
C8—H8*A*···O1^iii^	0.93	2.52	3.160 (3)	126

Symmetry codes: (i) −*x*, *y*−1/2, −*z*+1/2; (ii) −*x*−1/2, *y*−1/2, *z*; (iii) −*x*, −*y*+1, −*z*.

## References

[bb1] Bruker (2009). *APEX2*, *SAINT* and *SADABS* Bruker AXS Inc., Madison, Wisconsin, USA.

[bb2] Ghorab, M. M., Ragab, F. A., Alqasoumi, S. I., Alafeefy, A. M. & Aboulmaged, S. A. (2009). *Eur. J. Med. Chem* **45**, 171–178. 10.1016/j.ejmech.2009.09.039 19853327

[bb3] Sheldrick, G. M. (2008). *Acta Cryst.* A**64**, 112–122. 10.1107/S0108767307043930 18156677

[bb4] Spek, A. L. (2009). *Acta Cryst* D**65**, 148–155. 10.1107/S090744490804362X PMC2631630 19171970

